# Complete Genome Sequence of the Symbiotic Strain Bradyrhizobium icense LMTR 13^T^, Isolated from Lima Bean (Phaseolus lunatus) in Peru

**DOI:** 10.1128/genomeA.00146-18

**Published:** 2018-03-08

**Authors:** Ernesto Ormeño-Orrillo, Marco A. Rogel, Doris Zúñiga-Dávila, Esperanza Martínez-Romero

**Affiliations:** a Laboratorio de Ecología Microbiana y Biotecnología, Departamento de Biología, Facultad de Ciencias, Universidad Nacional Agraria La Molina, Lima, Peru; b Centro de Ciencias Genómicas, Universidad Nacional Autónoma de México, Cuernavaca, Morelos, Mexico

## Abstract

The complete genome sequence of Bradyrhizobium icense LMTR 13^T^, a root nodule bacterium isolated from the legume Phaseolus lunatus, is reported here. The genome consists of a circular 8,322,773-bp chromosome which codes for a large and novel symbiotic island as well as genes putatively involved in soil and root colonization.

## GENOME ANNOUNCEMENT

Bacteria commonly known as rhizobia provide biologically fixed nitrogen to their symbiotic legume partners. Thus, rhizobia can be used as biofertilizers to reduce the use of chemical fertilizers. The legume lima bean (Phaseolus lunatus) ranks second in cultivated area among beans of the Phaseolus genus only after the common bean (Phaseolus vulgaris). Lima bean is predominantly associated with slow-growing rhizobia of the Bradyrhizobium genus (1, 2). Previously, we have shown that four species of Bradyrhizobium are associated with lima bean in Peru (3, 4). Here, we present the complete genome sequence Bradyrhizobium icense LMTR 13, the type strain of one of those species that was isolated from a root nodule collected in an agricultural field in Ica, Peru (3).

DNA was sequenced using PacBio (RS II) and Illumina (HiSeq 2000) technologies. PacBio reads were *de novo* assembled by the single-molecule real-time (SMRT) Analysis pipeline using Hierarchical Genome Assembly Process (HGAP3) and the assembly polished using Quiver (5). Additionally, a hybrid assembly using Illumina and PacBio reads was obtained with SPAdes (6). Assembly reconciliation was performed by mapping reads over sequence differences and manually correcting possible errors or misassemblies. Gene prediction and annotation were performed using the NCBI PGAP (7).

The genome of LMTR 13^T^ was composed of single circular chromosome of 8,322,773 bp with a G+C content of 62%. The numbers of predicted coding sequences (CDSs) and tRNA genes were 7,456 and 53, respectively. A single rRNA operon was found in the genome. Functions could be assigned to 64% of the CDSs. Gene distribution by RAST functional categories revealed that the two most represented categories were those for the metabolism of carbohydrates and metabolism of amino acids that may be related to the soil/rhizosphere habitat of the bacterium. Genes for traits putatively involved in root colonization, such as those for polysaccharide synthesis, siderophore production, chemotaxis, and motility, were found in the genome. Auxin biosynthesis genes were found, suggesting plant growth-promoting activity for LMTR 13^T^. CDSs involved in resistance to osmotic, oxidative, heat, and cold stresses were also represented in its genome. A potential saprophytic lifestyle of LMTR 13^T^, and hence the ability to persist in the soil, was evidenced by the presence of many genes for the metabolism of aromatic compounds.

LMTR 13^T^ harbors a 985-kb symbiosis island (SI), representing 11.8% of its genome, which is the second largest rhizobial symbiotic compartment reported to date. The SI was flanked by an integrase gene and included three tRNA pseudogenes. Nodulation and nitrogen fixation genes were distributed along several SI regions, as well as genes involved in the biosynthesis of biotin and cobalamin, which may promote competitiveness for root colonization (8). An uptake hydrogenase gene cluster found within the SI may promote nitrogen fixation efficiency as in other rhizobia (9). Genes for a type III secretion system which may be related to interaction with its eukaryotic host (10) and for a type IV secretion system probably involved in the mobilization of the SI (11) were also found.

### Accession number(s).

The complete nucleotide sequence has been deposited in GenBank under the accession number CP016428.
